# Adaptation and validation of the Chinese version of the lung cancer screening health belief scales

**DOI:** 10.1186/s12889-022-13041-y

**Published:** 2022-03-30

**Authors:** Yu-an Lin, Lisa Carter-Harris, Jia-ni Yang, Xiu jing Lin, Fei fei Huang

**Affiliations:** 1 School of Nursing, Fujian Medical University, Fuzhou, China; 2 Memorial Sloan Kettering Cancer Center, New York, NY USA

**Keywords:** Lung Cancer Screening, Health Belief Model, Health beliefs, High-risk population, Reliability, Validity

## Abstract

**Background:**

Health belief is an important factor affecting lung cancer screening in high-risk population, but the research based on Chinese cultural background is still insufficient. Therefore, we adapted the English version of the Lung Cancer Screening Health Belief Scales (LCSHB) into the Chinese version (LCSHB-C) and examined its psychometric characteristics.

**Methods:**

After obtaining authorization from the original author, the LCSHB-C was adapted based upon Brislin's translation model. Using a variety of community-based recruitment methods, a total of 353 participants were recruited in Fuzhou, Fujian province, China to complete the questionnaires. We combined the classical test theory and item response theory to examine the psychometric properties of the LCSHB-C.

**Results:**

The Cronbach’s alpha for the four subscales ranged from 0.83 ~ 0.93. The content validity index for the four subscales was ranged from 0.87 ~ 1.0. Confirmatory factor analysis supported each subscale structure model fit well. Rasch analysis results further validated the reliability and validity of the four subscales. The person reliability and separation index of each subscale ranged from 0.77 to 0.87 and 1.83 to 2.63, respectively.

**Conclusions:**

The LCSHB-C is a reliable and valid instrument used to measure health beliefs related to lung cancer screening among those high-risk for lung cancer in China, which facilitates the development of lung cancer screening programs and promotes the "three early prevention strategies" of lung cancer (i.e.,early detection, early diagnosis and early treatment).

**Supplementary Information:**

The online version contains supplementary material available at 10.1186/s12889-022-13041-y.

## Background

Lung cancer is the most frequent cancer (2.1 million cases) and the leading cause of cancer-related deaths (1.8 million deaths) for both men and women worldwide [1]. The International Agency for Cancer Research reports that the incidence of lung cancer has been on the rise globally over the past ten years, especially in China. China accounts for the largest number of lung cancer patients in the world [2]. In fact, it is expected that the number of lung cancer patients in China will reach one million by 2025 [3]. Due to the asymptomatic nature of early lung cancer, individuals are often diagnosed at an advanced stage when the prognosis is poor or futile, with only a 12% to 16% relative five-year survival rate [4, 5]. However, if lung cancer can be identified at an early stage, the relative five-year survival rate increases to 60% to 70% [5, 6]. This evidence highlights the importance of secondary prevention for decreasing lung cancer-related mortality.

At present, low-dose computed tomography (LDCT) of the chest has been demonstrated to be a sensitive tool for the detection of early stage lung cancer [7]. Annual LDCT screening of the chest for high-risk individuals is recommended by the United States Preventive Services Task Force (Grade B) [8] and the Chinese government [9]. However, globally, screening with LDCT remains vastly underutilized, despite its proven morbidity and mortality benefit [5, 10, 11]. For example, from 2010 to 2015, less than 5% of high-risk individuals (i.e., individuals who currently or formerly smoked long-term) had been screened [10]. From 2018 to 2019, the participation rate of high-risk individuals in lung cancer screening in China was only 6.4% to 31.91% [12, 13].

Prior research has shown that factors related to low screening rates in screening-eligible individuals include psychological and cognitive variables (e.g., stigma, mistrust, fatalism, worry, fear, and low knowledge levels related to lung cancer and lung cancer screening), health beliefs (perceived risk, perceived benefits, perceived barriers, and self-efficacy), healthcare provider recommendation, and social and media exposure [13–15]. Of these factors, health beliefs are an important predictor of lung cancer screening behavior [5, 16]. In other words, the higher level of perceived benefits of, lower level of perceived barriers, and the higher level of self-efficacy, the more likely the individuals will screen for lung cancer screening [5, 11, 16], further supporting important constructions in the Conceptual Model for Lung Cancer Screening Participation [14].

Although research in this area remains early in its trajectory, evidence supporting knowledge and awareness about lung cancer screening remains low, and screening-eligible people continue to have a number of misconceptions regarding lung cancer screening, such as a scan is needed only if one is symptomatic or has not had a chest x-ray [15]. Previous research has also shown that individuals’ age, education level, annual income and health insurance were closely related to the perceived risk of lung cancer, perceived benefits of and perceived barriers to lung cancer screening [15, 17, 18]. However, little is known about the relationship of health beliefs to lung cancer screening participation among high-risk individuals in China, partly owing to the lack of psychometrically validated assessment tools.

Assessment and understanding of individual health beliefs about screening is a critical component to inform future efforts to promote the successful implementation of lung cancer screening programs and maximize the secondary prevention effect of LDCT screening. To our knowledge, the Lung Cancer Screening Health Belief scales (LCSHB) is one of the few instruments based on the health belief model that can be used to evaluate the perceived risk of lung cancer, perceived benefits of, perceived barriers to, and self-efficacy for lung cancer screening behavior [17]. Although the English version of the LCSHB is found to be psychometrically valid and reliable by classical test theory (CTT), further examination of the scale by item response theory (IRT) may provide more robust evidence [19]. Therefore, the main aim of the current study is to translate the English version of the LCSHB into Chinese and to investigate its psychometric properties with both CTT and IRT methods among the population at high risk for the development of lung cancer in China.

## Methods

### Participants and settings

From May 2020 to November 2020, participants were recruited using a variety of community-based recruitment methods, such as attaching our questionnaire QR codes on posters or contacting community staff to help post the information on bulletin boards, in Fujian province, China. Eligibility criteria mirrored the Chinese Expert Consensus on the Screening and Management of Lung Cancer [20] and Lung Cancer Screening National Comprehensive Cancer Network Clinical Practice Guidelines in Oncology Recommendation [21] for individuals eligible for lung cancer screening and included individuals (1) aged 55 to 74 years and individuals who currently smoke and have a 30 pack-year tobacco smoking history or individuals who used to smoke and have quit within the past 15 years; or (2) ≥ age 40 years and currently smoking with a 20 pack-year tobacco smoking history with one of the following risk factors: a) history of environmental or high-risk occupational exposure (e.g., exposure to asbestos, beryllium, uranium, radon); b) pulmonary disease (e.g., chronic obstructive pulmonary disease, diffuse pulmonary fibrosis or previous history of tuberculosis); c) previous malignant tumor history; d) family history of lung cancer; or e) long-term second-hand smoking exposure.

Participants with previous lung surgery, metal implants or devices in the chest or back, obesity and chest thickness, or diagnosed with lung cancer were excluded. The sample size was determined based on a subject-to-item ratio of 5–10:1 [22] by assuming a non-response rate of 15%, thus the final sample size was 360 potential participants to which the recruitment materials and survey were mailed.

### Design and procedures

**Fig. 1:**
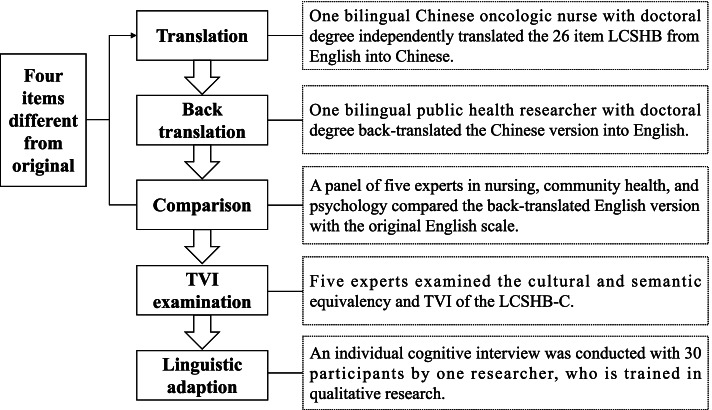
The cross-cultural translation of LCSHB-C. *LCSHB* Lung cancer screening health belief scales

After written permission was obtained from the original scale developer (Carter-Harris), we translated the LCSHB into the Chinese version (LCSHB-C) and then examined the psychometric properties of the LCSHB-C, which were found to be adherent to the COnsensus-based Standards for the selection of health status Measurement INstruments (COSMIN) checklist [23, 24]. We applied Brislin's translation model to the cross-cultural translation, which includes translation, back-translation, comparison, and linguistic adaption [25, 26], as showed in Fig. 1. When we compared the original and back-translated versions, we found four items in discrepancy and were re-translated and back-translated, including I7" I might put off having a lung scan because transportation would be a problem."(B7. I may postpone the lung scan because traffic will be a problem.); I14 "I might put off having a lung scan because I worry about feeling like a social outcast for smoking."(B14. I may postpone the lung scan because I am worried that I feel like a person abandoned by society because of smoking.); I15 "I might put off having a lung scan because I worry about being blamed for having smoked."(B15. I may postpone the lung scan because I worry about being blamed for smoking.); I28 "Compared to other people your same age who have never smoked, what would you say your risk of getting lung cancer is"(B28. Compared with other people who are the same age but not smoking, you think that your risk of lung cancer is).

In the stage of pilot testing, the interview used structured probes to uncover how participants interpreted items of the LCSHB-C to verify its comprehensibility and readability. Example probes included: “Tell me in your own words what this question is asking,” “How did you decide on your answer to this question?” and “What does [health beliefs about lung cancer screening] mean to you?” Interviews were audio recorded and transcribed verbatim. None of the participants reported confusion or incomprehension about any of the scale items. After this process, the LCSHB-C was ready for validation. In the survey, we adhered to the Strengthening the Reporting of Observational Studies in Epidemiology (STROBE) statement [27].

### Ethical considerations

The research was approved by the institutional review board of Fujian Medical University (Grant number:FM2020097), and written informed consents were obtained from all participants. Finally, a total of 353 participants enrolled and completed the 15-min survey and received a $10 gift card at completion. Participant anonymity was preserved in all cases.

### Data collection

Data were all collected through online surveys with QR codes on posters or bulletin boards by Wenjuanxi (the most popular online data collection platform in China, available at https://www.wjx.cn/). The study aim and data collection procedure were fully explained to the participants.

### Measures

#### LCSHB-C

The LCSHB consists of 35-items in total to evaluate an individuals' lung cancer screening health beliefs in the following four domains represented by the four subscales: (1) perceived risk of lung cancer, (2) perceived benefits of, (3) perceived barriers to, and (4) self-efficacy for lung cancer screening. All items in the perceived risk, perceived benefits, and perceived barriers subscales use a 4-point Likert scale (1 = strongly disagree to 4 = strongly agree) response option, and items in the self-efficacy subscale use the 4-point Likert scale of 1 = not at all confident to 4 = very confident. Items are all positively worded, and the total score is obtained by summing the scores of all items in each subscale. A higher total score on each individual subscale indicates higher perceived lung cancer screening health beliefs (perceived risk, perceived benefits, perceived barriers, and self-efficacy, respectively). The Cronbach’s alpha values ranged from 0.88 to 0.92 for the 4 subscales [17].

#### Lung cancer and screening knowledge questionnaire

This questionnaire was used to assess individuals' knowledge for lung cancer and screening, which was adapted from Carter-Harris' knowledge scale [18]. The questionnaire includes five questions with binary scoring (“correct” = 1, “wrong” = 0). The total score ranges from 0 to 5, and a higher total score indicates greater knowledge about lung cancer screening.

#### Socio-demographic questionnaire

We also collected participants' age, gender, marital status, educational level, monthly household income (yuan, RMB), residential location, religious belief, employment status, body mass index (BMI), health insurance, smoking status, family history of cancer, and frequency of lung cancer screening.

### Data analysis

Data analyses were conducted using SPSS 23.0 (IBM, Chicago, IL, USA) and WINSTEPS 3.75.0 (Chicago, IL, USA) with a *p*-value < 0.05 was considered significant. Missing values were replaced using the multiple imputation calculation [28].

- Cross-cultural validity: The 4-point COSMIN checklist [29] was used to measure whether the description of the translation scale well reflected the items in the original scale [23, 24].
- Content validity: Content validity was evaluated from the translation validity index (TVI) adapted from the content validity index (CVI) described by Tang and Dixon [30]. A four-point scale was employed to rate the translational relevance of each item on the four subscales (1 = “totally different”to 4 = “equivalent”). The item TVI (I-TVI) was calculated by dividing the number of experts with a relevance rating of 3 or 4 by the total number of experts. And the mean value of TVI for each item was the TVI of the total scale (STVI).
- Structural validity: Confirmatory factor analysis (CFA) in CTT and Rasch analyses were performed in combination to assess the construct validity of the scale. In the CFA, the best fitting model of each subscale was tested using the maximum likelihood method. Absolute and relative indices [31, 32], including normed χ2 (χ2/df) between 1.0 and 3.0, Root Mean Square Error of Approximation (RMSEA; < 0.08), Comparative Fit Index (CFI), Tucker-Lewis Index (TLI), and Normed Fit Index (NFI) > 0.9, were employed to evaluate the model's goodness of fit. In Rasch analysis, the unidimensionality assumptions were first checked by the first contrast of the residual to ensure that it was not higher than 2 [19] and then the rating scale model (RSM) was used to assess person/item separation reliability, person/item separation index, category probability curves, test information functions and person-fit statistics [33, 34]. Infit and outfit mean squares, as well as difficulty (location) for individual items were involved in Pearson’ s fit statistics [35]. Items were tested for the differential item functioning (DIF) by gender (male and female).
- Construct validity: We estimated the convergent validity of the four subscales of the LCSHB-C using Pearson's correlations, with expected significant positive correlations with the lung cancer and screening knowledge total score.
- Known-group validity: Known-group validity was performed by determining whether the subscale scores of the LCSHB-C could discriminate among participants with different frequency of lung cancer screening participation behaviors.
- Internal consistency: We used Cronbach's alpha to assess the internal consistency reliability of the four subscales [32].
- Floor/ceiling effect: Floor effects were evaluated by examining the percentage of the respondents that achieved the lowest possible scores. Ceiling effects were evaluated by examining the percentage of respondents that reached the highest possible score.

## Results

### Participants characteristics

A total of 353 valid questionnaires were returned out of the 360 questionnaires distributed (response rate, 98.06%). Descriptive statistics were reported using the medial and inter-quartile ranges due to the non-normality. For example, the age, smoking pack-years and BMI of the participants were 45 years (44.0, 52.0), 27.4 pack-years (27.4, 34.8) and 23.04 kg/m^2^ (21.0, 26.3), respectively. See Table 1 for a complete list of participant socio-demographic characteristics.

**Table 1:** The participant socio-demographic characteristics (*n* = 353)

Characteristic		n(%)
Gender	Male	284(80.45)
	Female	69(19.55)
Residential location	Urban	197(55.81)
	Suburban	59(16.71)
	Rural	97(27.48)
Educational level	Primary school degree or below	56(15.86)
	Middle school degree	78(22.10)
	Technical school/college degree	143(40.51)
	Bachelor’s degree or higher	76(21.53)
Religious belief	No religion	177(50.14)
	Christianity	44(12.46)
	Buddhism	124(35.13)
	Islamism	6(1.70)
	Others(e.g. Taoism)	2(0.60)
Employment status	worker	49(13.88)
	farmer	89(25.21)
	Administrative cadre	43(12.18)
	Science and technology、medical personnel or teacher	49(13.88)
	Individual, business, enterprise or service personnel	103(29.18)
	Retired	13(3.68)
	Housewife	6(1.70)
	Others ^a^	1(0.28)
Occupational exposure ^b^	Yes	208(58.92)
	No	145(41.08)
Medical insurance	Self-paid (uninsured)	42(11.90)
	Provincial medical insurance	73(20.68)
	Municipal medical insurance	99(28.05)
	New agricultural cooperative medical insurance	138(39.09)
	Others(e.g. medical insurance for urban employees)	1(0.28)
Monthly household income (yuan, RMB)	< 1000	30(8.50)
	1000–2999	87(24.65)
	3000–4999	123(34.84)
	> 5000	113(32.01)
Smoking status	current smoker	267(75.64)
	Former smoker (now quit)	86(24.36)
Smoking status of family members	current smoker	286(81.02)
	Former smoker (now quit)	67(18.98)
History of lung disease	Yes	58(16.43)
	No	295(83.57)
Metal implants or devices in your chest or back	Yes	100(28.33)
	No	253(71.67)
Suffering from cancer	Yes	12(3.40)
	No	341(96.60)
Family history of cancer	No	273(77.34)
	Unclear	65(18.41)
	Yes	15(4.25)
Frequency of lung cancer screening	Never	142(40.23)
	Every year	147(41.64)
	Uncertain	64(18.13)

^a^blacksmith

^b^Such as asbestos, chromate, coke oven efflux, arsenic, chloromethyl ether, radon and its progeny, radiation, silica, beryllium, uranium, radon and other substances

### CTT Validity Testing of the LCSHB-C

#### Cross-cultural validity

The process of translation and the sample size (≥ 150) met the requirements of “adequate” in the COSMIN checklist [29].

#### Content Validity of the LCSHB-C

Five experts were consulted (All with doctoral degree and have well working experience on nursing and public health). Expert consultation demonstrated that S-TVI ranged from 0.87 to 1.0 for four subscales.

#### Structural Validity of the LCSHB-C

**Fig. 2:**
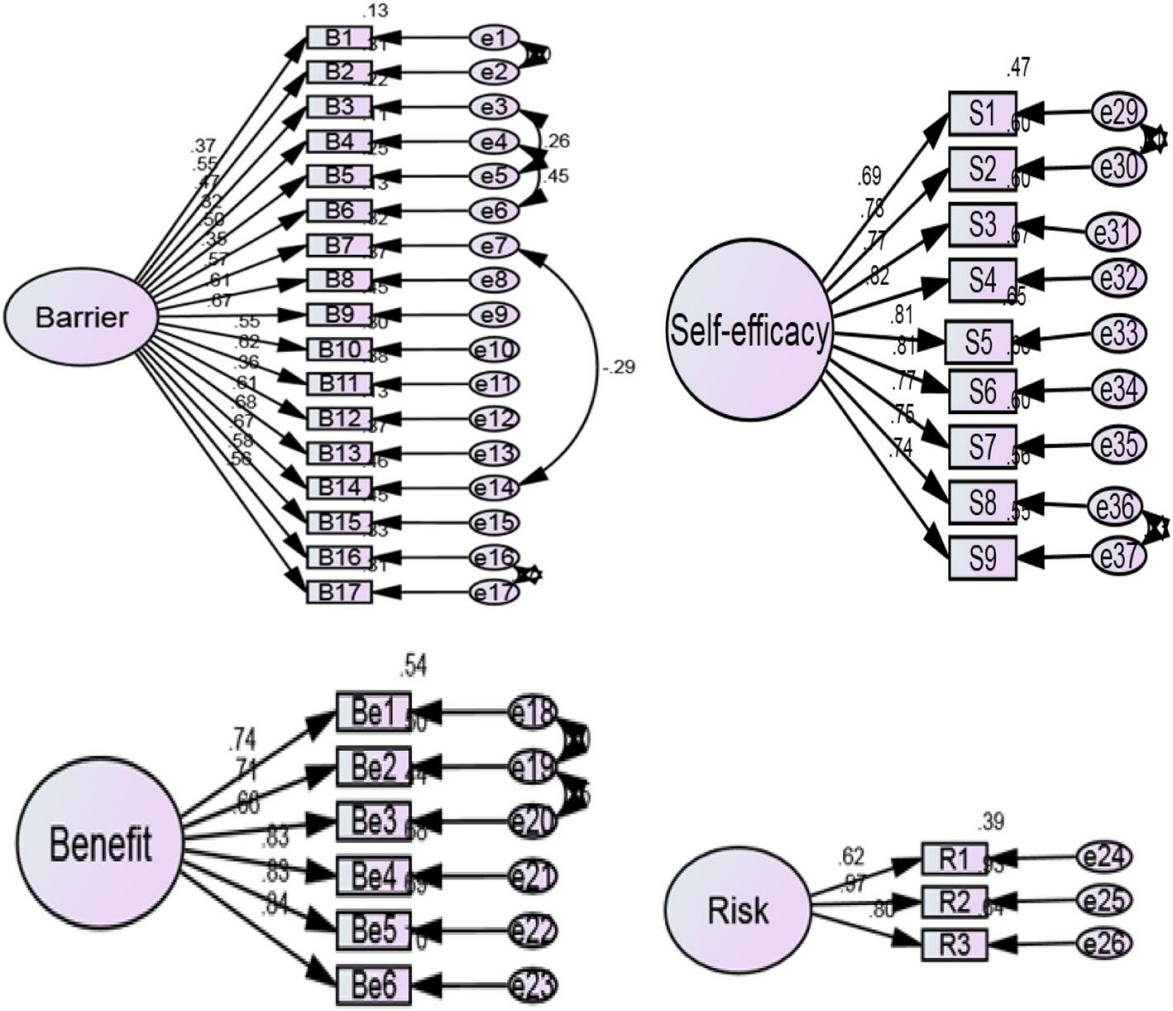
The factor structure of each subscale of the Chinese version of Lung cancer

As shown in Fig. 2, the single-factor models of perceived barriers to lung cancer screening subscale, perceived risk of lung cancer subscale, perceived benefits of lung cancer screening subscale, and self-efficacy for lung cancer screening subscale were fit well and confirmed by CFA. The model fit indices of the LCSHB-C subscale models are shown in Table 2.

**Table 2:** The model fit indices of the LCSHB-C scale and subscale models

	χ2	χ2/df*	RMSEA	CFI	TLI
The perceived barriers of LCS subscale model	318.03	2.79	0.07	0.88	0.87
The perceived benefits of LCS subscale model	18.56	2.65	0.07	0.99	0.98
The perceived risk of LCS subscale model	3.13	0.78	0.00	0.99	0.99
The self-efficacy for LCS subscale model	83.25	3.33	0.08	0.97	0.96

*LCS* lung cancer screening, *p < 0.05

#### Construct Validity of the LCSHB-C

Pearson’s correlation analysis showed that the subscale score of perceived benefits of lung cancer screening and self-efficacy for lung cancer screening was significantly positively correlated with the lung cancer and screening knowledge score (*r* = 0.203, 0.154, 0.230, *p* < 0.01), respectively. However, both the perceived barriers to lung cancer screening score and the perceived risk of lung cancer score was not correlated with the total knowledge score in this sample.

#### Known-group validity of the LCSHB-C

We found that there were no significant differences between screeners and non-screeners for perceived barriers and perceived risk scores, but screeners had significantly higher perceived benefits, and self-efficacy (see Table 3).

**Table 3:** Scale means examining differences between participants who have Screened for lung cancer and unscreened

	Scale Range	Overall (*n* = 353) Mean (SD)	Screeners (*n* = 211) Mean (SD)	Non-screener (*n* = 142) Mean (SD)	T value (*p* value)
Total perceived barriers scores	17–68	40.10(8.48)	40.21(8.13)	39.92(9.01)	0.32(0.75)
Total perceived benefits scores	6–24	17.80(3.88)	18.24(3.83)	17.14(3.89)	2.63(0.01*)
Total perceived risk scores	3–27	6.93(2.29)	7.11(2.28)	6.67(2.29)	1.78(0.08)
Total self-efficacy scores	9–36	26.44(6.16)	27.64(5.71)	24.64(6.38)	4.62(0.00*)

*SD* standard deviation, *p < 0.05

#### Floor/ceiling effect

As shown in Table 4, the four subscales have a lack of floor or ceiling effects, that is, the occurrences of the lowest and highest possible four subscale scores were both below 15%.

**Table 4:** The floor/ceiling effect analysis of four subscales

Subscale	Score ranges	Lowest score (%)	Highest score(%)
Perceived barriers	17–68	1.4%(5/353)	1.7% (6/353)
Perceived benefits	6–24	2.3%(8/353)	10.8%(38/353)
Perceived risk	3–12	11.6(41/353)	3.7%(13/353)
Self-efficacy	9–36	2.5%(9/353)	11.3%(40/353)

#### Rasch Analysis of the LCSHB-C

In the Rasch analysis, the unidimensionality assumption of each subscale was supported by the first contrast of the residual ranging from 1.7 to 2.0 (less than 2). As shown in Table 5, the infit and outfit mean squares for each item ranged from 0.63 to 1.43. The differential item functioning was not found when evaluated by gender. No evidence of disordered thresholds was found in the category probability curves, as the category calibration increased in an orderly way (see Appendix A). We also found the item reliability and separation index was 0.82(2.37), 0.81(2.14), 0.94(3.88), and 0.72(2.00), and person reliability and separation index was 0.85(2.41), 0.82(2.15), 0.77(1.83), 0.87(2.63) for the barriers, benefits, risk and self-efficacy subscales, respectively. Regarding the test information functions, both subscales gathered information most precisely when Ө ranged from 0 to 2.0 (see Appendix B).

**Table 5:** The Rasch analysis of the LCSHB-C

Subscale	Item	Item difficulty ^a^	Infit MNSQ	Outfit MNSQ	DIF contrast by gender^b^
Perceived barriers to lung cancer screening	B1	0.10	1.26	1.43	0.82
	B2	0.00	0.90	0.90	1.72
	B3	0.02	0.97	1.04	1.71
	B4	-0.17	1.17	1.25	1.35
	B5	-0.01	0.95	0.94	0.06
	B6	-0.03	1.03	1.04	0.55
	B7	-0.02	1.00	1.01	0.28
	B8	-0.01	0.94	1.00	0.01
	B9	-0.01	0.91	0.92	0.89
	B10	-0.06	0.93	0.96	1.69
	B11	-0.14	0.85	0.87	0.03
	B12	0.01	1.17	1.33	2.15
	B13	0.07	0.88	0.87	0.42
	B14	0.04	0.93	0.92	0.04
	B15	0.01	0.81	0.81	2.1
	B16	0.01	1.02	1.06	1.19
	B17	0.21	1.26	1.40	1.77
Perceived benefits of lung cancer screening	Be1	-0.17	1.12	1.04	0.57
	Be2	0.02	0.94	0.88	0.52
	Be3	0.16	1.14	1.11	2.38
	Be4	0.04	0.92	0.83	0.39
	Be5	-0.08	0.93	0.83	0.18
	Be6	0.03	0.92	0.83	0.01
Perceived risk of lung cancer screening	R1	-0.68	1.38	1.38	0.30
	R2	0.14	0.66	0.63	0.42
	R3	0.54	0.91	0.89	0.01
Self-efficacy for lung cancer screening	S1	0.32	1.21	1.26	1.66
	S2	0.10	0.89	0.89	0.00
	S3	-0.11	1.10	1.12	0.15
	S4	-0.14	0.90	0.88	0.59
	S5	-0.12	0.92	0.90	0.75
	S6	0.05	0.87	0.86	1.87
	S7	-0.11	0.96	0.93	0.40
	S8	-0.03	1.06	1.02	0.41
	S9	0.05	1.06	1.04	0.37

*MNSQ* mean square

^a^Measured in logit; positive item logit indicates that the item requires a lower visual ability than the mean of the items and is an easier item; a negative item logit indicates that the item requires a higher visual ability than the mean of the items and is a more difficult item

^b^male compared with female

#### Reliability of the LCSHB-C

The Cronbach’s alpha for the subscale of perceived barriers to lung cancer screening, perceived risk of lung cancer, perceived benefits of lung cancer screening, and self-efficacy for lung cancer screening were 0.88, 0.90, 0.83, and 0.93.

## Discussion

This study adapted and validated the LCSHB scales to Chinese, following standard translation and cultural adaptation guidelines [36]. Our psychometric evaluation, based on the CTT and Rasch analysis, showed that the LCSHB-C (perceived risk, perceived benefits, perceived barriers, self-efficacy) can provide sufficient validity (cross-cultural validity, structural validity and construct validity), satisfactory internal consistency reliability, without no floor/ceiling effect. The reliable and valid LCSHB-C will contribute to a more accurate evaluation and in-depth understanding of the levels and types of health beliefs for lung cancer screening among individuals at risk for the development of lung cancer who speak Chinese. Improving health beliefs in a population at high-risk for the development of lung cancer can ultimately improve the lung cancer screening decision-making process and subsequent screening behavior [14]. Thus, the Chinese version of LCSHB scales can now inform tailored intervention development as well as programmatic efforts to increase lung cancer screening uptake in the high-risk Chinese population.

This study demonstrated the applicability of the LCSHB scales for the high-risk population of lung cancer in China and its good reliability and validity based on CTT and Rasch analysis. Interestingly, we found that the Chinese and English versions of the LCSHB shared the same factor structure [17]. Confirmed with the expanded health belief model [37], our findings indicated that the LCSHB scales can adequately measure perceived risk of lung cancer, perceived benefits of, perceived barriers to, and self-efficacy for lung cancer screening among the high-risk population at high risk for the development of lung cancer in China.

Similar to prior work [17], we found that screeners had higher health beliefs related to lung cancer screening (i.e., perceived benefits, self-efficacy) than non-screeners, which extends the work of Carter-Harris and colleagues by supporting consistency between the original work in an English-speaking American population and a Mandarin-speaking Chinese population. The significance of this work lies in the ability to validly measure health beliefs in the high-risk Chinese population to identify potentially modifiable individual-level factors on which to intervene in future lung cancer screening outreach programs. In addition, construct validity of the LCSHB-C was also supported by the significant positive correlations with the total lung cancer screening knowledge scale score, which is consistent with the previous work [18]. Finally, internal consistency reliability was supported with all 4 subscales noting a Cronbach's alpha more than 0.70 [32].

Apart from the traditional CTT methods, we also examined the construct validity of LCSHB-C by Rasch analysis, with the results showing that the category rating scale of the LCSHB-C was in good operation. Besides, LCSHB-C with acceptable measurement precision is sensitive to differentiating between high and low levels of health beliefs associated with LCS according to the results of the combination of a good person-separation index (> 2) and person reliability (> 0.8) [34].

### Limitation

As with all studies, this study is not without limitations. First, participants were recruited from one province in China which may impact generalizability to all Chinese individuals and that future work that examines health beliefs across geographically diverse areas of China is warranted. Second, the inclusion criteria of this study mainly referred to the Chinese Expert Consensus on the Screening and Management of Lung Cancer [20], which differed from the inclusion criteria of the original scale in two aspects. The younger age of the participants might be related to the fact that online surveys were mostly conducted by younger groups. On the other hand, the smoking history was relatively light, which might be related to the greater health awareness of the included participants, with nearly 56% of the people surveyed coming from urban areas. Therefore, the sample may be not representative enough. Future studies can not only adopt the method of the on-site survey to better understand the difference of participants between the two routes of completion but also pay more attention to an older group or those in rural and suburban areas, such as recruiting through recommendation from community healthcare providers or adopting stratified sampling. Third, some psychometric characteristics of the LCSHB-C could be assessed further, such as test–retest reliability. Moreover, the sensitivity of the LCSHB-C subscales was not assessed. Therefore, future longitudinal or experimental studies are warranted. A further refinement of the scale based on a larger representative sample will produce more stable parameter estimations and robust results.

## Conclusions

The Chinese version of LCSHB scale comprised of four subscales is a sufficiently valid and reliable tool for assessing health beliefs for lung cancer screening among the populations at greatest risk for the development of lung cancer in China. The scales can also contribute to a better understanding of how health beliefs in the context of lung cancer screening operate within high-risk populations who make the decision to screen, or not, for lung cancer in China. Finally, the LCSHB-C can also inform the development of lung cancer screening outreach programs by providing a psychometrically valid tool to evaluate the effects of such programs.

### Relevance for clinical practice

Evidence has consistently indicated that individual health belief about screening is a common barrier to the uptake of the secondary prevention of lung cancer screening—especially among those with a high disease burden related to lung cancer in China [5]. Community nurses and local healthcare providers can use the LCSHB-C to accurately measure perceived risk of lung cancer, perceived benefits of, perceived barriers to, and self-efficacy for lung cancer screening behavior. Furthermore, this scale can also facilitate the development of lung cancer screening outreach programs and evaluate the effects of future interventions. Additional research with more representative samples is needed to further examine the screening utility of this scale. It will also be important to determine the cut-off value for the LCSHB-C subscales (low, middle, and high levels of the four health beliefs for lung cancer) and to compare the health belief for screening among high-risk individuals of lung cancer globally.

## Supplementary Information

**Additional file 1.**

## Data Availability

The datasets used and/or analyzed during the current study are available from the corresponding author on reasonable request.
