# Supplementary material for: Adaptation and validation of the Chinese version of the lung cancer screening health belief scales
Source: BMC Public Health. 2022 Mar 30;22:620. doi: 10.1186/s12889-022-13041-y (PMC8969234; doi:10.1186/s12889-022-13041-y)
Supplement: Supplementary file 1 — Additional file 1. [file 12889_2022_13041_MOESM1_ESM.docx]

Appendix A The Category probability curves for the Chinese version of Lung cancer screening health belief subscales


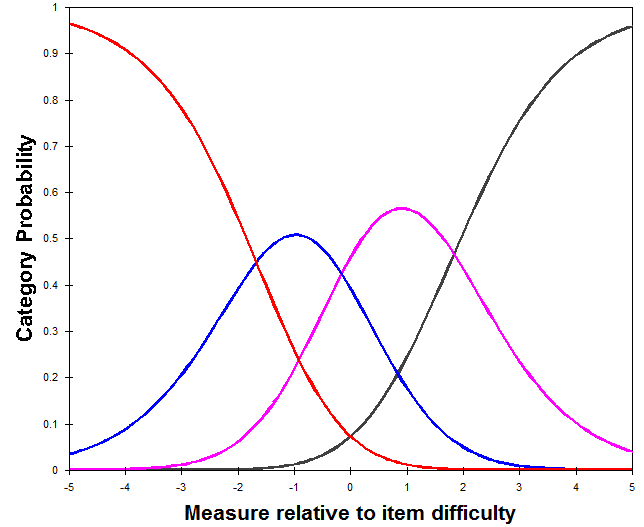

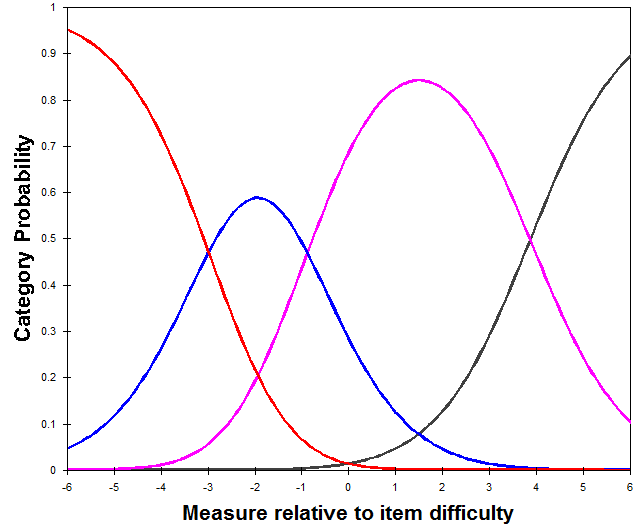


B1 of barrier subscale ^a^ Be1 of benefit subscale ^a^


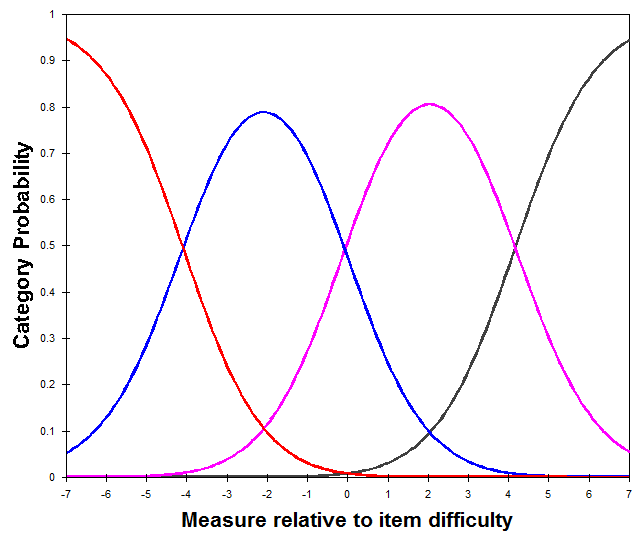

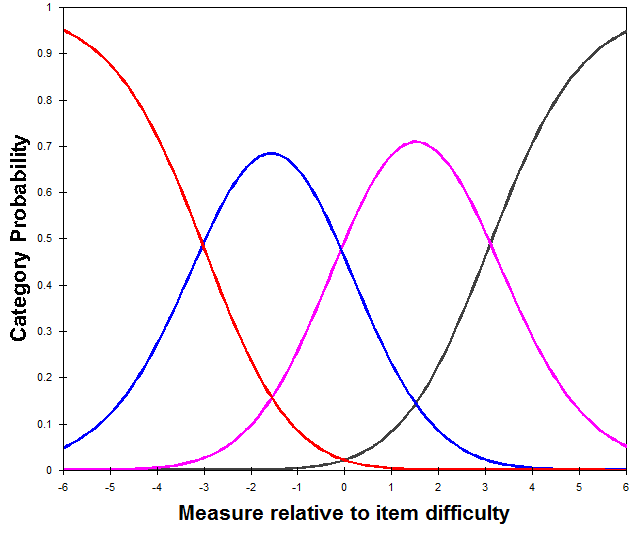


R1 of risk subscale ^a^ S1 of self-efficacy subscale ^b^

^a^ The four curves from left to right represent 4 response categories (1 = *Strongly disagree*; 2 =*disagree*; 3 = *agree*; 4 =*strongly agree* ).

^b^ The four curves from left to right represent 4 response categories (1 = *Not at confident*; 2 =*Not too confident*; 3 = *Somewhat confident*; 4 = *very confident* ).

Appendix B The test information function for the for the Chinese version of Lung cancer screening health belief subscales


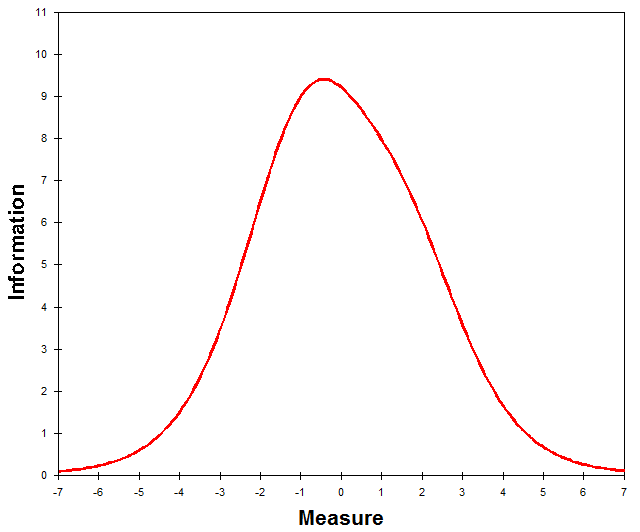

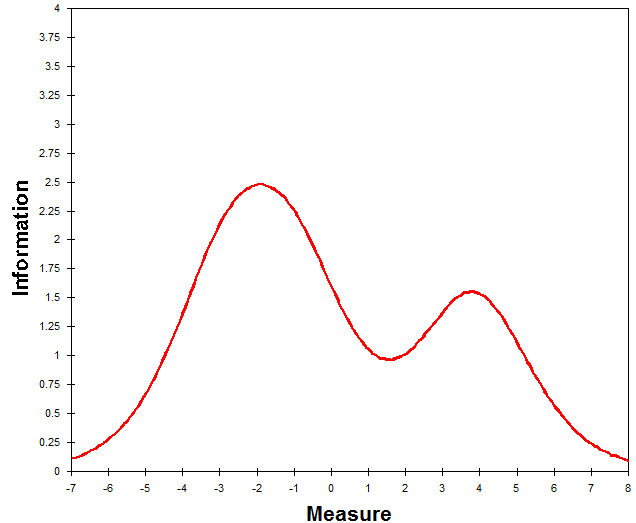


Barrier subscale Benefit subscale


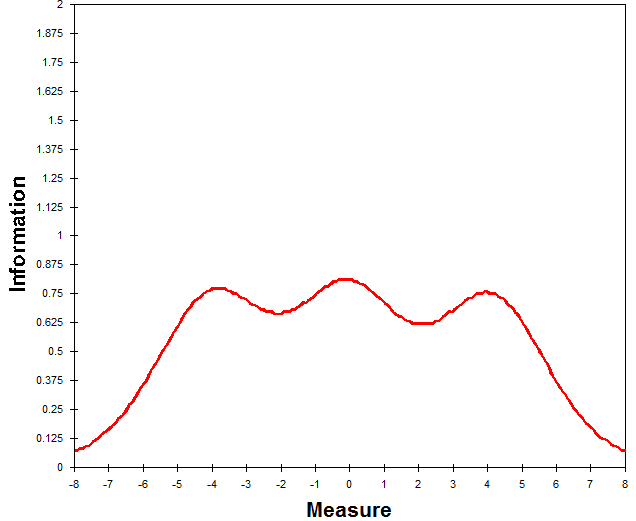

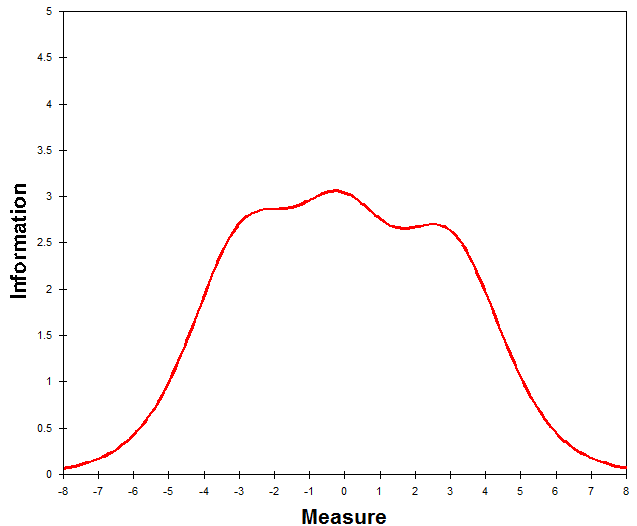


Risk subscale Self-efficacy subscale
